# *TEMGYM Basic*: transmission electron microscopy simulation software for teaching and training of microscope operation

**DOI:** 10.1107/S1600576723005174

**Published:** 2023-07-14

**Authors:** David Landers, Ian Clancy, Dieter Weber, Rafal E. Dunin-Borkowski, Andrew Stewart

**Affiliations:** a Department of Physics, University of Limerick, Limerick, Munster V94 T9PX, Ireland; b Department of Chemistry, University College London, 20 Gordon Street, London WC1H 0AJ, United Kingdom; c Ernst Ruska-Centre for Microscopy and Spectroscopy with Electrons (ER-C), Forschungszentrum Jülich, Wilhelm-Johnen-Straße, Jülich, North Rhine-Westphalia 52428, Germany; DESY, Hamburg, Germany

**Keywords:** Python, computer programs, simulation, virtual microscopes, transmission electron microscopy, alignments, interactive software, ray tracing, *TEMGYM Basic*

## Abstract

An interactive simulation of a transmission electron microscope (TEM) called *TEMGYM Basic* is developed here, which enables users to understand how to operate and control an electron beam without the need to access an instrument. *TEMGYM Basic* allows users to familiarize themselves with alignment procedures offline, reducing the time and money required to become a proficient TEM operator. In addition to teaching the basics of electron beam alignments, the software enables users to create bespoke microscope configurations and develop an understanding of how to operate the configurations without sitting at a microscope. *TEMGYM Basic* also creates static ray diagram figures for a given lens configuration. The available components include apertures, lenses, quadrupoles, deflectors and biprisms. The software design uses first-order ray transfer matrices to calculate ray paths through each electron microscope component, and the program is developed entirely in Python to facilitate compatibility with machine-learning packages for future exploration of automated control.

## Abstract 2

*TEMGYM Basic* is a Python-based ray-tracing tool for novel experiment visualization and an interactive learning environment for using transmission electron microscopes.

## 1. Introduction

Transmission electron microscope (TEM) characterization methods are becoming increasingly important within crystallographic studies to elucidate structures, resolve the impact of defects, understand stress–strain relationships and identify grain boundaries. In particular, the adoption of 3D electron diffraction and 4D scanning transmission electron microscopy methods have become more widespread, along with the use of classical TEM imaging techniques (Gemmi & Lanza, 2019 ▸; Krysiak *et al.*, 2021 ▸) to characterize and determine the relationships between structure and properties.

As electron microscopy characterization techniques become widespread, there is an increasing need for scientists with TEM experience to acquire high-quality data, the key step to which is the ability to accurately align the TEM. However, to the authors’ knowledge, there are no software packages available that interactively simulate the electron beam trajectory through a TEM, enabling users to learn the alignment procedures and visualize the outcome as it would appear on the microscope ‘green screen’ in real time. The software *TEMGYM Basic* aims to fill this gap and enable users to learn the operation of a TEM offline in a risk free environment, as well as teaching new users how to prepare a microscope for experimentation. Several online resources, including https://rodenburg.org (Rodenburg, 2023 ▸) and *MyScope* (Whiting *et al.*, 2022 ▸), offer excellent introductory materials explaining the steps involved in aligning a microscope. However, they do not contain interactive visualization of the electron beam path in the microscope or the ability to customize the lenses present in the instrument, both of which are possible within *TEMGYM Basic*. Some examples of interactive software (*e.g.* Nagatini, 2019 ▸) give users a fundamental insight into how the beam path moves through a column of the TEM but only include lens components. *TEMGYM Basic* extends currently available methods to inform and teach users the basics of aligning a TEM, bringing an interactive aspect to material that is typically static ray diagrams.

A significant challenge for new users is understanding how to translate simplified 2D figures in a user manual (which often assumes knowledge a novice user may not possess) to the practical steps necessary to align the microscope. *TEMGYM Basic* bridges the gap between documentation for the alignment procedures and the practical experiment *via* interactive visualization of the beam movements in the microscope column as the controls are adjusted, and how this influences the resulting beam spot on the detector. For example, the model can show how a quadrupole corrects astigmatism, how apertures occlude the beam, or how a pair of deflectors operate together to tilt and shift the beam. These provide unique insight to help new users connect theory from an alignment manual with the instrument operation.

The program combines matrix multiplications and geometry functions representing electron microscope components with a graphical user interface (GUI), which facilitates user control of the electron ray path and visualization of the resultant spot on the detector in real time. The program utilizes visualization packages such as *PyQtGraph* (Moore & Campagnola, 2022 ▸) and *Matplotlib* (Hunter, 2007 ▸) to visualize the ray paths and the electron beam spot. The interactive nature enables the user to experiment with the beam path, understand the behaviour of the different lenses, apertures and biprisms, and explore how control of the instrument affects the alignment. The simulation uses first-order approximations of each component within the simulation, meaning that the behaviour of the electron beam will only match a real electron microscope when the beam is close to the optic axis.

Furthermore, the program can visualize any arrangement of electron microscope components from those available within the software (lenses, apertures, dipoles, quadrupoles, biprisms and deflector pairs), and users can choose to place components where they prefer on the optic axis to create any TEM setup they desire.

Electron microscopes [TEMs and scanning electron microscopes (SEMs)] are sensitive and expensive instruments, and training time to teach and learn how to operate these instruments is an expensive and limited commodity. Another similar resource developed to help TEM facilities prepare users to use electron microscopes more effectively is *MyScope*. A recent study from the developers of *MyScope* has seen an increase in core user knowledge and a reduction in training time required when people use *MyScope* as part of their training process. The aim with *TEMGYM Basic* is to continue to enhance a student’s learning environment and augment existing training and educational tools with an interactive electron beam alignment platform. Although this work focuses on modelling a TEM, the software can also model an SEM setup, which we demonstrate in Section 5.

## 2. Software structure

**Figure 1:**
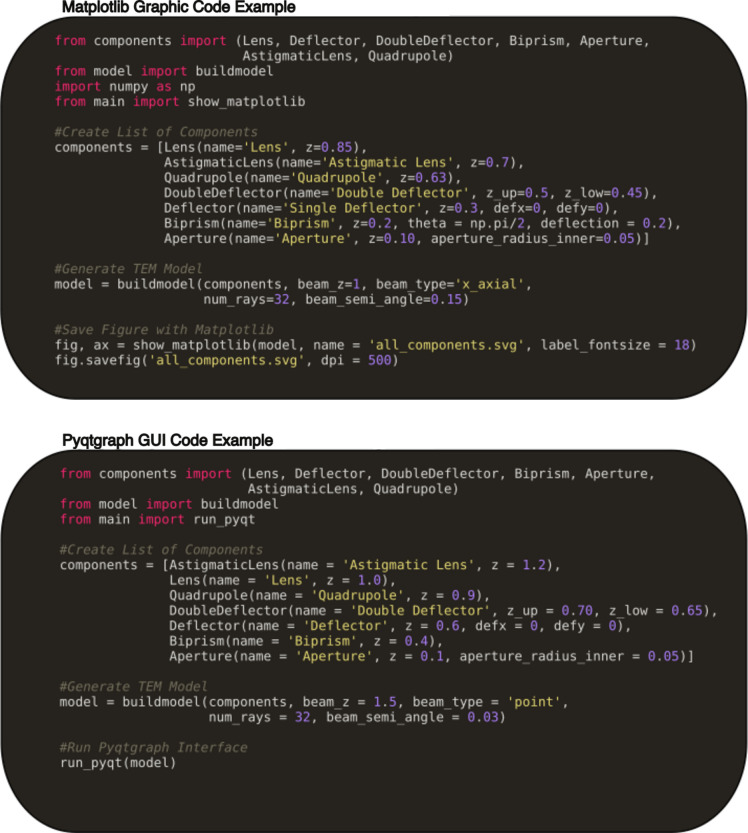
Example Python code layout for creating a model in the software using either *Matplotlib* or *PyQtGraph*.

*TEMGYM Basic* is written entirely in Python. The software is contained in a series of six scripts that create the GUI, calculate the ray paths and model the 3D components of the TEM. In addition, nine example tutorials introduce how to use the software. These include how to use the 3D interactive simulation and how to output 2D figures. Users can run a compiled executable available for Windows, Mac and Linux, or modify the example scripts to create a custom microscope model and run the Python scripts from the command line. The design philosophy allows users with a basic knowledge of Python to construct example models by populating a list with their desired components (Fig. 1 ▸). The software then reads this list and automatically generates the appropriate interactive 3D model and GUI interface [via the package *PyQtGraph* (Moore & Campagnola, 2022 ▸)] for each TEM component specified by the user. This virtual TEM model can be updated in real time via the interactive GUI, allowing users to practise the base alignments qualitatively.

Where possible, the computational and GUI elements of the software are separated, enabling a user to create a 3D interactive model or to generate 2D figures for talks and papers via *Matplotlib*, which makes a static graphic of the ray paths through their model electron microscope. It is also possible to use other graphics packages to render the 2D graphics with minor modifications to the code.

We have fixed several design conventions within the code. The model assumes that the detector plane sits at 0 (the bottom of the image) and the gun sits at some positive value above 0 (*i.e.* the gun sits above the detector) with the electron source pointing down the *z* direction. It is a user-defined choice of where to place the gun on the optic axis. All components are then given a positive *z*-position value between the gun and the detector and propagate rays from the gun to the detector plane.

The model generates the beam spot by counting which rays have landed inside the detector, and there are two visualization modes available: (i) to count the pixels that have a ray impinging on them or not, which will generate a beam spot; or (ii) to count the number of rays at a given pixel, assigning an intensity value for the number of rays impinging upon each pixel. The latter mode is useful when a biprism is included in the experimental setup, enabling visualization of where the beam interference fringes would occur during an experiment.

The user can also modify the initial distribution of electrons, of which there are three choices:

(*a*) A point source that spreads electrons out in a uniform circular distribution from a singular point.

(*b*) A radial source whereby electrons begin uniformly distributed around a point in a circle and can also have an initial angle in *x* or *y*.

(*c*) An axial source, which creates rays aligned with the *x* and *y* axes.

### 2.1. Graphical user interface

The GUI comprises a single main window divided into three separate areas: a 3D model of the electron microscope components and current beam path, a detector window showing the resulting beam spot, and a GUI window enabling users to interact with each element and observe the response. A parameter table that shows the current settings for all components in a separate list is also optionally displayed in the program window.

**Figure 2:**
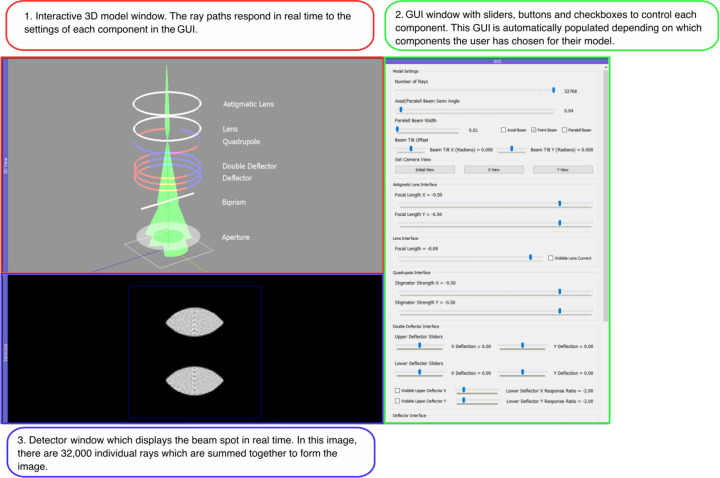
A screenshot of the three main components of the interactive window paired with coloured captions detailing the purpose of each component.

*PyQtGraph* GUI elements, such as buttons, sliders and checkboxes, control the program settings and microscope parameters along with the behaviour of each element that a user has specified in their list of components (see Fig. 2 ▸). For instance, a slider can precisely control the focal length of a lens, or its focal length can be oscillated back and forth by selecting the ‘wobbler’ checkbox. Controlling the program in this way allows users to recreate the rotation-centre alignment in a TEM, which requires a user to oscillate a lens’s focal length (Fig. 6).

A vital aspect of the software program is the adaptable nature of the GUI. Once a user has populated a list with their desired electron microscope components, the code automatically populates each interface in the model appropriately:

(1) It creates a 3D model of each component in its corresponding position in the interactive 3D viewer.

(2) The code automatically populates the *PyQtGraph* interface with the correct GUI element for each component that the user has specified. This adaptable nature creates an interactive GUI for each unique electron microscope model. It makes it easy for users of the software with minimal programming experience to create custom models for exploration and teaching.

(3) The model traces rays through each component, visualizes the 3D ray path and displays the corresponding beam spot on the screen.

### 2.2. Matrix description of software

**Figure 3:**
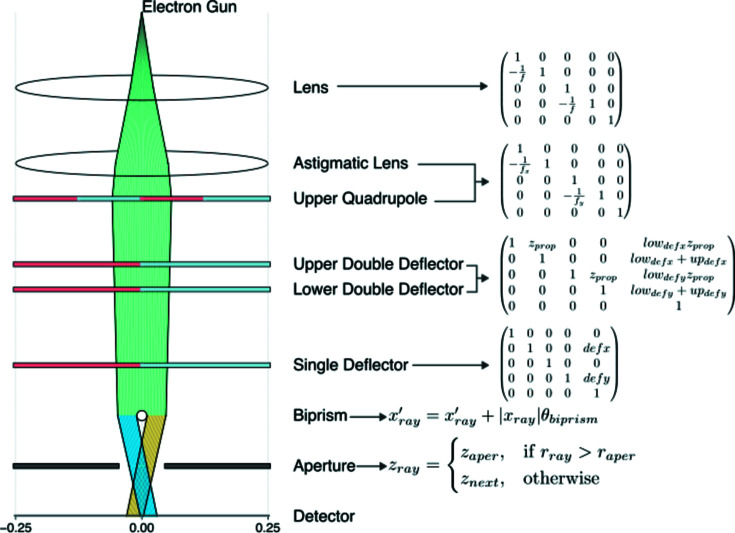
Ray transfer matrices and appropriate function that models the first-order behaviour of each component in the software.

The software uses linear ray transfer matrices to propagate each electron ray in straight lines from each component. Ray transfer matrices readily pair with the array programming library *NumPy* (Harris *et al.*, 2020 ▸) and allow *TEMGYM Basic* to propagate thousands of rays through many components in real time interactively as the user adjusts the control menu. We have modified the traditional 2 × 2 component transfer matrix in one dimension to a 5 × 5 matrix for the two-dimensional case, following the convention set out by Mansell *et al.* (2006 ▸). The fifth column in the matrix facilitates a deflection parameter, so the user can control the deflection angle of a beam as it passes through a deflector. Each ray is represented by a 5 × 1 ray matrix, with the elements representing the *x* position, *x* slope, *y* position, *y* slope and a value of 1 in the final row. The value of 1 in the final row facilitates the multiplication of the deflection variable in the 5 × 5 transfer matrix of deflector components (see Fig. 3 ▸). The ray transfer matrix formulation is particularly convenient as it enables the program to propagate thousands of rays via a single matrix multiplication for components such as a lens, deflector or quadrupole.

However, matrix multiplication methods cannot propagate rays through an aperture or biprism, so these components require an alternative approach. Concerning an aperture, logic statements check whether a ray should pass through or if it is excluded from further propagation. As for a biprism, rays deflect in opposite directions depending on which side of the biprism they fall upon. We model this behaviour via a vectorized *NumPy* sign function acting on each ray. This function checks which side of the biprism a ray has landed on and multiplies the deflection amplitude by the appropriate sign value, creating the effect of a biprism splitting the electron beam within the context of ray matrices. Finally, we combine the *z* positions of each component with the *x*–*y* positions from each ray matrix and generate a matrix describing each ray path from one component to the next, which the 3D GUI can then display.

## 3. Program limitations

*TEMGYM Basic* is a tool designed to help users grasp the underlying concepts of an electron microscope more clearly and generate publication-quality diagrams that capture the essence of an experimental setup. In an experimental instrument, the scale of the microscope (metres) and the scale of the beam path (micrometres to nanometres) are orders of magnitude apart. Representing the microscope’s scale and the electron ray paths within a typical computer screen is therefore impossible for visualization reasons. Therefore, we have chosen to represent distances in the software at a scale suitable for 3D representation of the components and electron ray paths. However, with minor modifications to the software, it is possible to rescale the model to match the dimensions found in the instrument, and a user could rescale the beam path to make it visible while calculating the exact first-order beam location behind the scenes. We also ignore the rotation of the electron beam inside a magnetic lens, and the ability to include the aberrations of each component is not provided in this iteration of the software.

## 4. Example alignment models

To further facilitate the usability of the software, we have created several example alignment models that recreate vital steps in the alignment process of a TEM. The examples are available as precompiled binaries for Windows, Mac or Linux. Each example demonstrates a basic principle necessary to align a TEM.

### 4.1. Beam tilt/shift

The lens and deflector components implemented in the model enable users to simulate the beam-tilt/shift alignment, one of the critical steps in the alignment of an electron microscope.

The tilt/shift alignment requires that users set the current to a pair of beam deflectors such that the beam will perform a pure tilt or pure shift over the sample. This arrangement of two deflectors frequently appears inside a TEM, and the model contains a component called a ‘Double Deflector’, which creates the appropriate sliders to tilt and wobble the beam.

**Figure 4:**
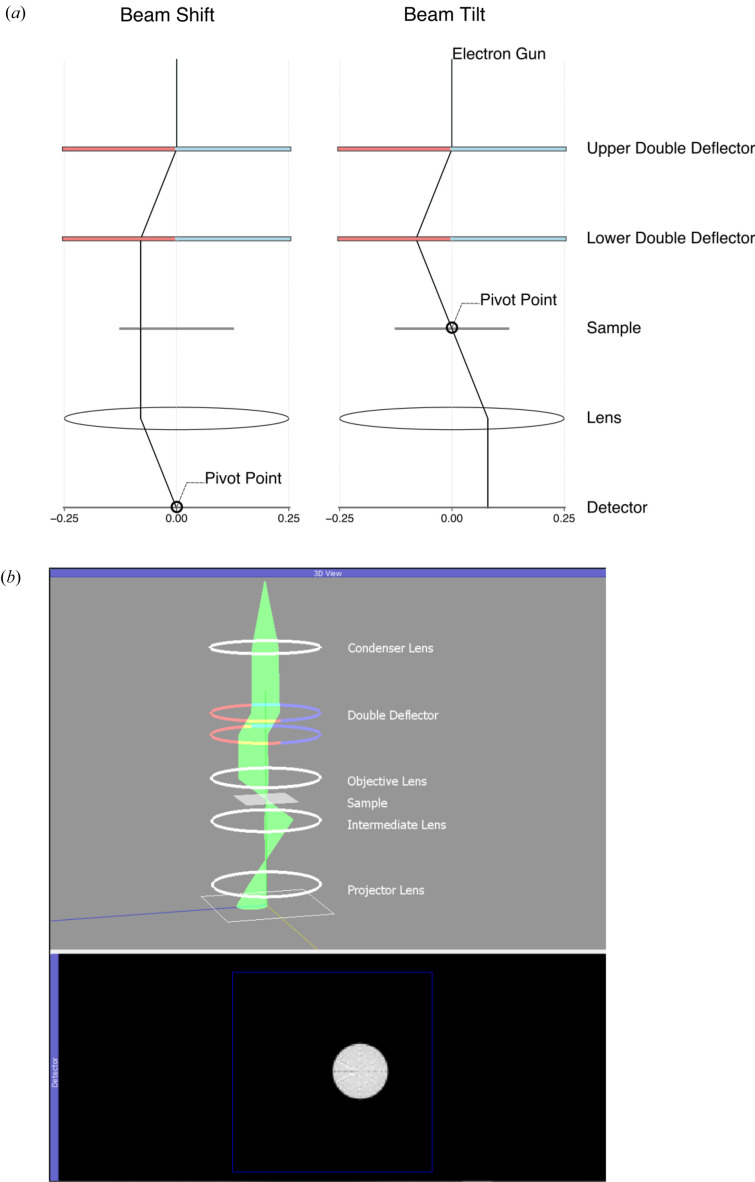
(*a*) A beam-shift/tilt alignment model schematic drawing, as seen in a typical TEM alignment manual, created in the software via *Matplotlib*, and (*b*) an interactive version created using *PyQtGraph*.

When performing the tilt/shift alignment, the instrument controller wobbles the current to an upper deflector, which in turn causes the beam to oscillate around a ‘pivot point’. In practice, users adjust the lower deflector’s response to the upper deflector via a deflector-ratio parameter to obtain the correct pivot point for beam tilt or beam shift. The consequence of adjusting the deflector ratio is to move the beam’s pivot point up or down the column (Fig. 4 ▸).

For beam shift and beam tilt, the appropriate beam pivot is at two different locations in the column: the back focal plane and the front focal plane, respectively. By adjusting the strength of an intermediate lens, it is possible to select either the front (image) or back focal (diffraction) plane, and a user can visualize the beam on the detector to see if they have set the deflector ratio correctly for beam tilt or beam shift.

In the beam-shift alignment, the user adjusts the deflector ratio such that the beam remains parallel to the optic axis and will shift across the sample. This alignment is correct when the beam spot remains stationary in the back focal plane because all rays on the objective lens are parallel to the optic axis.

During the beam-tilt alignment, the user adjusts the deflector ratio until the beam tilts around a single point on the detector. The tilt alignment is complete when the tilting beam converges to a single spot in the front focal (image) plane, regardless of which direction the beam is tilting.

### 4.2. Condenser astigmatism

**Figure 5:**
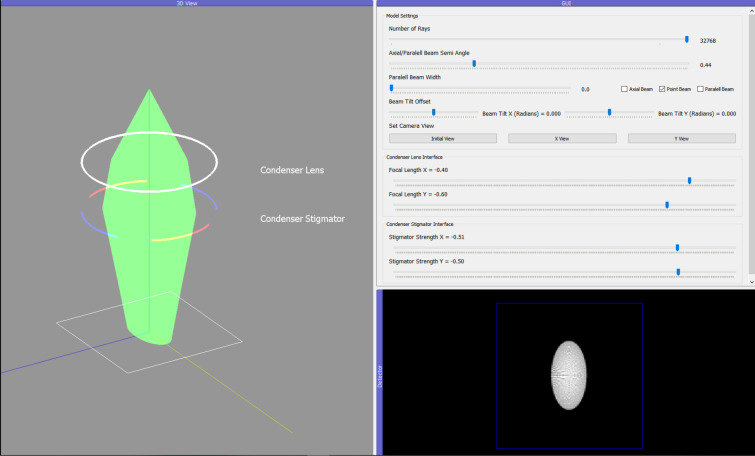
An interactive condenser-astigmatism alignment model in the software.

When training new users, we have experienced that the condenser-astigmatism alignment is one of the most challenging to understand. Therefore, to alleviate some of the difficulties users experience when learning the condenser-astigmatism alignment, we also created a simplified 3D visualization inside *TEMGYM Basic* (see Fig. 5 ▸).

Due to limitations in the manufacturing processes of magnetic lenses, they are not rotationally symmetric. These imperfections create variations in focal lengths along different lens axes, which is called astigmatism. A pair of quadrupole magnets in electron microscopes counteract this aberration by correcting for the asymmetric focal length of the lens. Within *TEMGYM Basic*, the stigmator component comes with two sliders to adjust the strength of its focusing effect on the *x* and *y* axes, which corrects for astigmatism in a corresponding lens. The user can ascertain that the astigmatism is corrected by visually inspecting the beam spot on the detector and checking that it is round instead of elliptical.

### 4.3. Rotation centre

**Figure 6:**
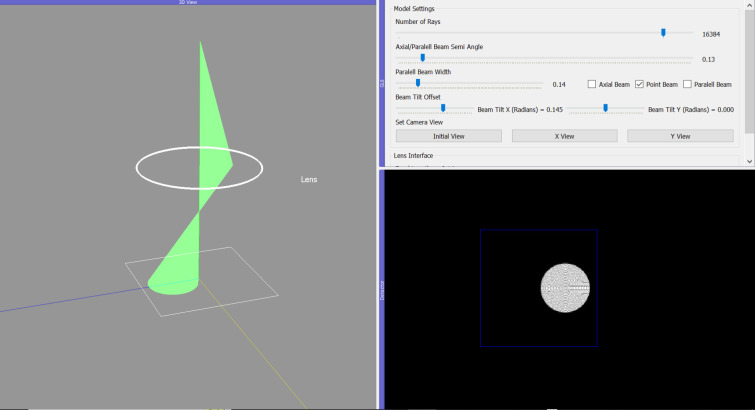
A misaligned beam in the rotation-centre alignment.

Users perform the rotation-centre alignment to ensure that the beam is centred on the optic axis of the microscope. The rotation-centre alignment is demonstrated in *TEMGYM Basic* using a single lens and a tilted beam (see Fig. 6 ▸). The lens strength is first ‘wobbled’. The spot position will change in the *x*–*y* plane of the detector (or green screen) if the beam is not aligned with the optic axis. However, if the beam position stays the same, then optimal alignment has been achieved.

### 4.4. Condenser aperture

**Figure 7:**
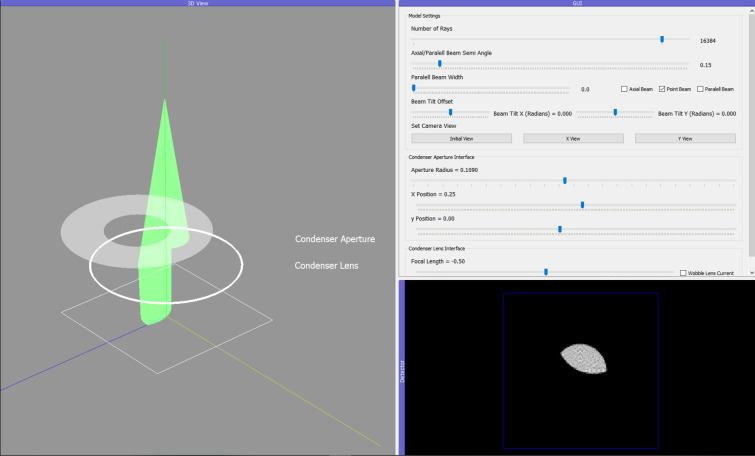
A misaligned condenser-aperture alignment, which is occluding the beam.

The aperture-alignment procedure involves varying the focal length of the condenser lens through a crossover (the point at which the beam converges to its smallest point), enabling visualization of the aperture alignment relative to the optical axis. If the beam position shifts in the *x*–*y* plane on either side of the crossover, the aperture must be adjusted halfway between the two beam centres. The process repeats until the beam position is the same on either side of the crossover (see Fig. 7 ▸).

### 4.5. Biprism

**Figure 8:**
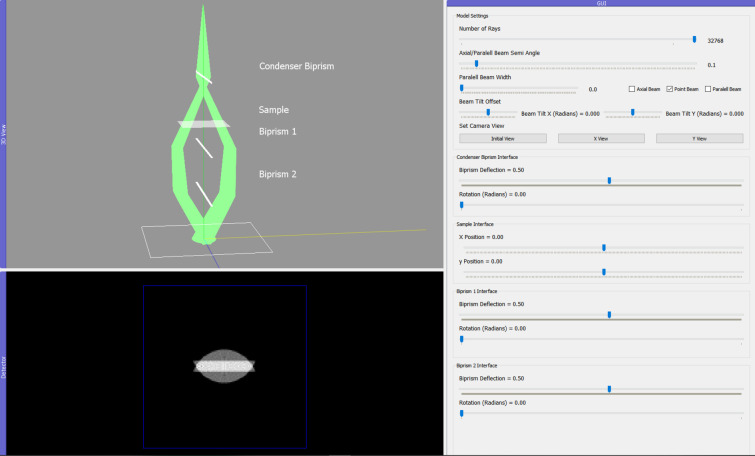
A split biprism experimental setup with three biprisms.

The biprism has been successfully used to demonstrate Young’s double slit phenomena in an electron microscope and experimentally verify the quantum nature of the electron (Tavabi *et al.*, 2019 ▸; Merli *et al.*, 1976 ▸). It is also a necessary component for conducting holography experiments, enabling the reconstruction of the phase of an electron image and the measurement of the magnetic properties of materials. The simplified model in *TEMGYM Basic* implements a biprism that can converge or diverge the beam and demonstrates the basic principles of how a biprism manipulates the beam to obtain the conditions for an interference experiment (see Fig. 8 ▸).

Tanigaki *et al.* (2012 ▸) demonstrated a split condenser biprism experimental setup, allowing a user to perform an interference experiment on any part of a sample using three biprisms instead of one. The *TEMGYM Basic* model can easily recreate an interactive version of this experimental setup.

## 5. TEM and SEM showcase

**Figure 9:**
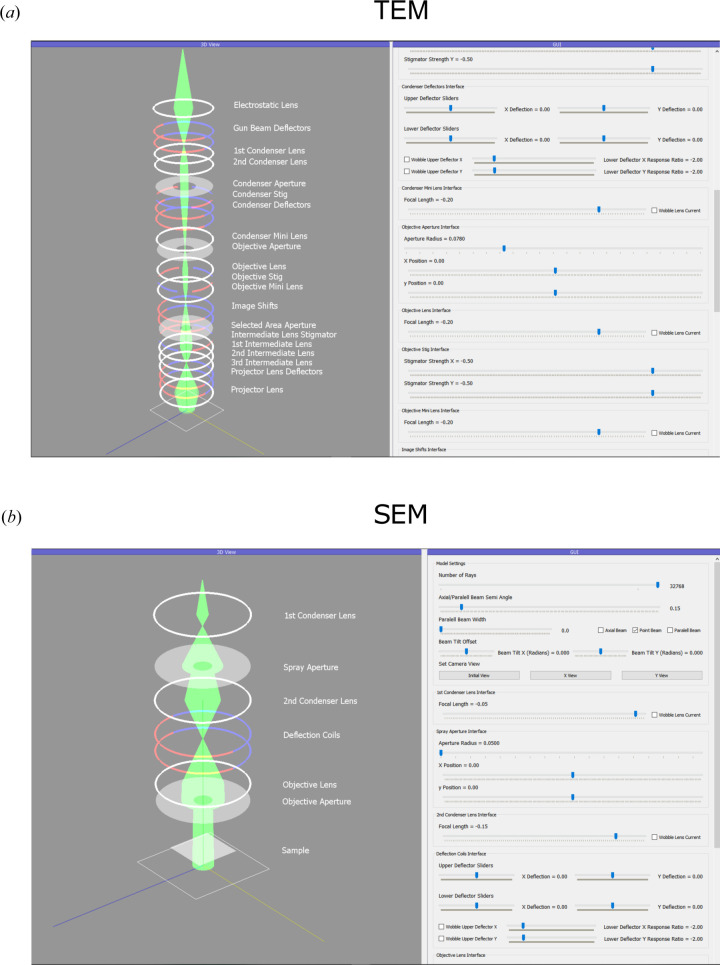
(*a*) A realization of a JEOL 2010F schematic drawing in the software (Sarney, 2004 ▸). (*b*) A model of a generic SEM schematic drawing in the software (Steff, 2010 ▸). The sample in the TEM schematic drawing would be located inside the objective lens but is not indicated on the schematic drawing.

The components implemented in *TEMGYM Basic* are sufficient to realize qualitative models of TEM and SEM schematic drawings. Microscope-alignment manuals typically show basic schematic drawings of their electron microscope; we have chosen to implement a schematic drawing displayed in a JEOL 2010F manual (Sarney, 2004 ▸) and a generic SEM schematic drawing (see Fig. 9 ▸). In the JEOL 2010F manual, a graphic displays the order of components inside the column. However, there are no other details, such as the exact location of each component or the first-order imaging parameters. Thus, we cannot place components accurately into the model to produce a digital twin of the microscope, nor can we replicate the first-order properties of lenses or deflectors since that information is proprietary. However, if one did have access to these details, one could easily recreate first-order beam-path diagrams of their electron microscope. Nevertheless, a *TEMGYM Basic* user can gain insight into how varying the parameters of one component inside a microscope can influence the beam trajectory within the microscope, be that an SEM or a TEM; for example, by varying the strength of the condenser lenses, one can vary the beam’s size on the detector.

SEMs contain similar components to a TEM, such as lenses, deflectors and apertures, and *TEMGYM Basic* can quickly generate an interactive model of a generic SEM schematic drawing (Steff, 2010 ▸). The *TEMGYM Basic* microscope models will enable users to more comprehensively understand their manipulation of the electron beam within a microscope and how each component controls and manipulates the electron beam inside a TEM or SEM. In addition, given the expense of time and money involved in training an electron microscope user, *TEMGYM Basic* provides a cost-effective training tool independent of the microscope for practising how to operate and align the instruments.

## 6. Publication-quality ray-path diagrams with *Matplotlib*

**Figure 10:**
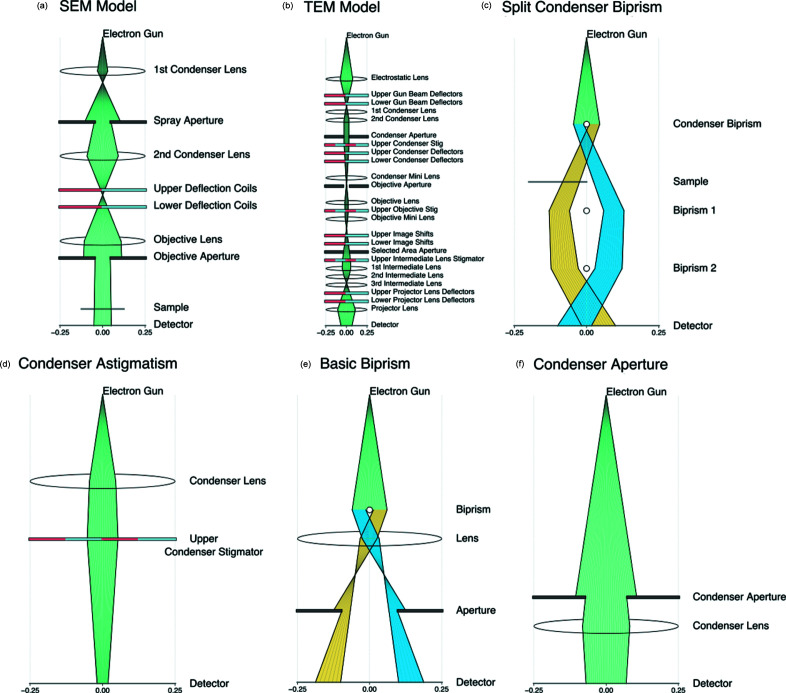
Six *Matplotlib* ray diagrams of various microscope schematic drawings generated via *TEMGYM Basic*.

Users are not limited to displaying a ray path with the GUI that we have developed in *PyQtGraph*. The code can also produce static publication-quality ray-tracing diagrams in combination with the popular Python plotting library *Matplotlib* (Hunter, 2007 ▸) via a single function call (see Fig. 10 ▸). Furthermore, users can modify the *Matplotlib* code to generate the type of plot they wish to make or save the file as scalable vector graphics (SVG) and manually edit each component in an editor of their choice. Finally, we expect users who wish to understand the first-order properties of a new experimental setup or educate others on the essential operation of a microscope will find this feature valuable.

## 7. Conclusions

We have presented *TEMGYM Basic*, an interactive flexible easy-to-use software package that aims to reduce the barriers that new users experience when learning to align an electron microscope. The software utilizes a first-order simulation of the electron ray path and a GUI program composed of four distinct parts: a 3D visualization of the microscope components and the path of the electron beam through the column, a visualization of the electron beam on the fluorescent screen or detector, an interactive controller for each TEM component, and the ability to output the alignment as a 2D ray diagram. Additionally, the modular nature of the *TEMGYM Basic* code allows users to create arbitrarily complex arrangements of microscope components, enabling users to model how such a design would operate to a first approximation.

In the future, this software could also act as groundwork to generate real-time virtual-reality models of ray paths associated with the first-order behaviour of components inside an electron microscope. Upcoming work built upon the foundation of this software will focus on an advanced version that aims to model and visualize the behaviour of realistic TEM components.

*TEMGYM Basic* has been written in Python and is freely available on GitHub under a GPL3 licence (https://github.com/AMCLab/TemGymBasic), and the associated documentation is available at https://temgymbasic.readthedocs.io/en/latest/. *TEMGYM Basic* is designed to be lightweight and to run on any operating system with low computational power.
